# Supplementary material for: Quantitative analysis of genome packaging in recombinant AAV vectors by charge detection mass spectrometry
Source: Mol Ther Methods Clin Dev. 2021 Aug 26;23:87–97. doi: 10.1016/j.omtm.2021.08.002 (PMC8476707; doi:10.1016/j.omtm.2021.08.002)
Supplement: Document S1. Supplemental materials and methods, Tables S1 and S2, and Figures S1 and S2 [file mmc1.pdf]

OMTM, Volume *23*

Supplemental information

Quantitative analysis of genome packaging in recombinant AAV vectors by charge detection mass spectrometry

Lauren F. Barnes, Benjamin E. Draper, Yu-Ting Chen, Thomas W. Powers, and Martin F. Jarrold

Analysis of Alkaline Gels and Comparison to CDMS results

BioRad Image Lab 5.2.1 (version 6.0.1.34) software was used to perform a densitometry analysis of the alkaline gels. The gel and CDMS signals can be divided into three main subclasses: the high mass peak or band at close to the packaging capacity, the GOI peak or band, and the low intensity, low mass tail that extends to the empty capsid in some of the CDMS spectra. The analog of the CDMS low mass tail was absent or small in the gels and so we focused on the high mass band and the GOI band for the gels. The relative integrated intensities are summarized in Table S1. We caution that the features often overlap and the locations of the dividing lines between them is somewhat subjective.

The gels and CDMS measurements have different sensitivities. The signals from bands on a gel are proportional to the number of DNA bases present while for CDMS the signal is proportional to the number of molecules. Thus the gels are more sensitive to longer DNA molecules and this explains why the low mass tail evident in the CDMS measurements is absent or small in the gels. To enable a quantitative comparison between the gels and the CDMS measurements we corrected for the sensitivity difference by multiplying the CDMS signals by the DNA mass given by the measured mass minus the mass of the empty particle. We then integrated the signals in the GOI peaks and the high mass peaks. The fraction of the signal in the GOI peak (the integrated signal for the GOI peak divided by the sum of the integrated signals the high mass and GOI peaks) is shown in Table S2 which also includes the corresponding fraction of the GOI band from the gels.

Figure S1 shows a plot of the GOI fraction obtained from the gels versus the GOI fraction obtained from the adjusted CDMS measurements. The dashed line in the figure shows the result where the GOI fraction from the gels and CDMS measurements are identical. The relative intensities from the gels are in most cases slightly higher than the relative intensities from the CDMS measurements. The two measurements are completely different so the good agreement between them is gratifying.

Table S1. Table showing the percentages of the signal in the high mass peak or band, GOI peak or band, and the low mass tail for the GOIs studied here. For the gels the low mass tail was small or missing and it is not included. Results are shown for alkaline gels with (+) and without (−) DNase I treatment.

	Alkaline Gel (−)		Alkaline Gel (+)		CDMS		
GOI	High Mass Band	GOI Band	High Mass Band	GOI Band	High Mass Peak	GOI Peak	Low Mass Tail
CMV-CRE	33	67	38	62	26	64	10
CMV-GFP	39	61	48	52	32	49	19
CMV-mcherry	37	63	34	66	22	68	9
CAG-GFP	35	65	39	61	20	58	22
CAG-mcherry	32	68	38	62	25	62	13
EF1a-GFP	25	75	26	74	18	58	25
CBA-GFP	-	100	-	100	-	72	28
CMV-SaCas9	-	100	-	100	-	52	48

Table S2. Table showing the fractions in the GOI peaks or bands for the different GOIs. The fractions were determined from the integrated intensity of the GOI peak or band divided by the sum of the integrated intensities of the high mass and GOI peaks or bands. Results are shown for the alkaline gel, with (+) and without (−) DNase I treatment. The results for the CDMS measurements have been adjusted as described above to account for the sensitivity of the gels to DNA length.

GOI	Fraction in GOI Peak or Band		
	Gel (−)	Gel (+)	CDMS
CMV-CRE	0.67	0.62	0.57
CMV-GFP	0.61	0.52	0.48
CMV-mcherry	0.63	0.66	0.63
CAG-GFP	0.65	0.61	0.63
CAG-mcherry	0.68	0.62	0.62
EF1a-GFP	0.75	0.74	0.72

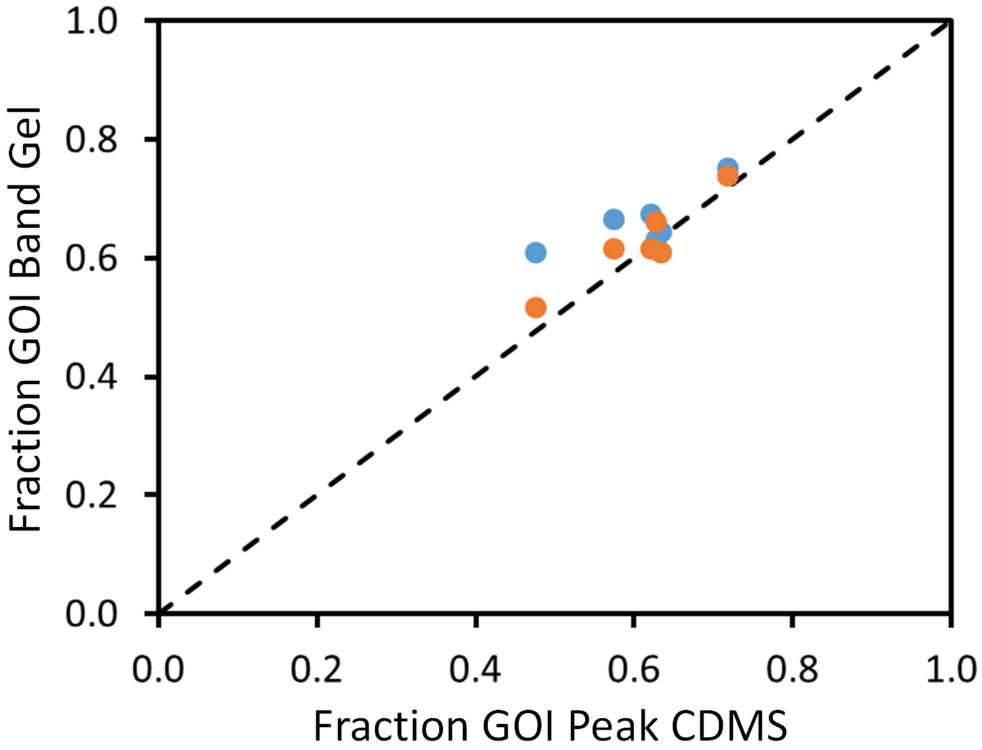

Figure S1. Plot of the relative intensity of the GOI peak or band determined from the gels against the same quantity determined by CDMS. The orange points are for gels with DNase I the blue points are without. The dashed line shows the result where the gels and CDMS give the same fractions.

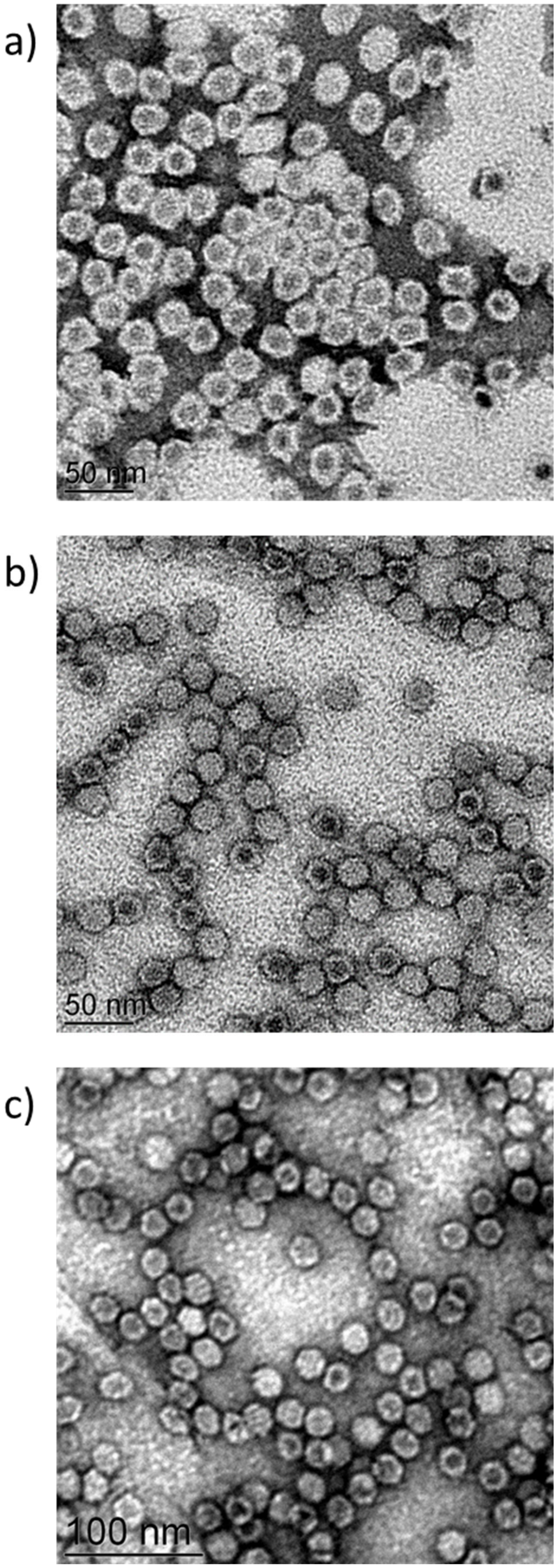

Figure S2. Representative TEM images for Virovek vectors. a) empty AAV8; b) CMV-SaCas9 (4.84 kb); and c) CMV-CRE (2.22 kb). In a) stain has penetrated most of the particles (they have a darkened center) indicating that they are empty. CDMS measurements for this sample indicate that all the particles are empty. A few particles in the image, particularly near the top, are not stained, presumably because the stain did not penetrate. In b) most of the particles have not picked up stain suggesting that they are full. The CDMS measurements for CMV-SaCas9 show a peak for the GOI near the packaging capacity and a broad low mass tail (comprising around 48% of the signal) that extends almost to the mass of the empty capsid. The stained particles in this case must have partial genomes. In c) there is a heterogeneous mixture where some particles are not stained (full), some are lightly stained (partial), and some are heavily stained (empty). The CDMS measurements for CMV-CRE show a peak at close to the packaging capacity, a peak for the GOI at intermediate masses and low mass tail that extends to the empty capsid mass. Overall, the CDMS measurements provide a more robust characterization of the genome content than negative stain EM.
