# Supplementary material for: Quantitative analysis of genome packaging in recombinant AAV vectors by charge detection mass spectrometry
Source: Mol Ther Methods Clin Dev. 2021 Aug 26;23:87–97. doi: 10.1016/j.omtm.2021.08.002 (PMC8476707; doi:10.1016/j.omtm.2021.08.002)
Supplement: Document S2. Article plus supplemental information [file mmc2.pdf]

Molecular Therapy
Methods & Clinical Development
Original Article

Quantitative analysis of genome packaging in recombinant AAV vectors by charge detection mass spectrometry

Lauren F. Barnes,[1] Benjamin E. Draper,[1] Yu-Ting Chen,[2] Thomas W. Powers,[2] and Martin F. Jarrold[1]

[1]Chemistry Department, Indiana University, 800 East Kirkwood Avenue, Bloomington, IN 47405, USA; [2]Pfizer, Inc., BioTherapeutics Pharmaceutical Sciences, Analytical R&D, 875 Chesterfield Parkway West, Chesterfield, MO 63017, USA

Recombinant adeno-associated virus (rAAV) has emerged as an important gene therapy vector with many clinical trials currently in progress. Analytical characterization and quantitation of particle content remain challenges in both the development and production of rAAV vectors. In this study, charge detection mass spectrometry (CDMS) and gel electrophoresis are used to characterize the DNA content of recombinant AAV8 (rAAV8) vectors with a wide range of target genome sizes. We show that the differences between the masses of empty particles and particles with the genome of interest (GOI) are correlated with the expected genome mass. A small systematic deviation (around 2%) is attributed to the packaging of counterions along with the DNA. In addition to the GOI, a broad distribution of heterogeneous DNA is packaged. The distribution peaks are close to the packaging capacity of the rAAV8 vectors. There is also evidence for the co-packaging of small DNA fragments along with the GOI. Finally, we present evidence that incubation at an elevated temperature can reduce the heterogeneity of the packaged DNA. Taken together, these results show that CDMS is a viable tool for characterization of the packaged genome.

INTRODUCTION

Adeno-associated virus (AAV) is a small, non-enveloped, single-stranded DNA virus, around 25 nm in diameter.[1,2] Wild-type AAV (wtAAV) packages approximately 4.7 kb[3] and requires a helper virus, such as adenovirus or baculovirus, to replicate.[4] Recombinant AAV[5,6] is a promising gene therapy vector, with two AAV-based treatments currently US Food and Drug Administration (FDA) approved and many ongoing clinical trials.[7,8] The AAV capsid contains three proteins (VP1, VP2, and VP3) generated by alternative initiation. All three share the VP3 sequence, VP2 adds an N-terminal domain, and VP1 consists of the VP2 sequence plus a further N-terminal domain.[3] For capsids derived from HEK293 cells the ratios of VP1–VP3 are around 1:1:10.[9–12] The AAV capsid has a total of 60 capsid proteins arranged in pseudo-icosahedral symmetry.[13–15]

The packaging capacity of AAV vectors is generally considered to be less than around 5 kb.[16,17] There have been several reports of larger transgene delivery.[18,19] However, further studies showed that this was due to either incompletely assembled particles or to the intracellular reassembly of gene fragments.[20–23] Single-stranded genomes of both + and − strands are packaged with equal frequency.[24] The detailed mechanism of genome packaging is still the subject of debate.[25] Packaging occurs primarily from the 3′ end into a preassembled capsid through a pore at a 5-fold symmetry axis.[26,27] When the genome exceeds the packaging capacity, packaging proceeds until the capsid is full and then unpackaged DNA is truncated by cellular nucleases.[22] Both wtAAV particles and recombinant AAV (rAAV) vectors can package incomplete genomes even when the genome length is less than the packaging capacity.[28–30] Several strategies have been developed to express transgenes that exceed the packaging capacity.[31,32]

The preparation of clinical grade AAV vectors requires methods to generate, purify, and characterize them.[33] The traditional production method is through DNA plasmid transfection of HEK293 cells.[34] AAV production using insect *Sf9* cell lines (baculovirus helper) allows for highly scalable suspension growth cultures.[35–38] Baculovirus synthesized proteins undergo post-translational modifications and have similar biological activity to proteins synthesized in mammalian cells. However, there remain issues with the generation of fully functional mammalian AAV viruses in insect cell lines.[39]

Characterizing rAAV vectors is challenging because their size places them well beyond the range of traditional analytical methods and technologies, including high-resolution mass spectrometry, that are usually used to characterize and confirm the identity of small-molecule or traditional biotherapeutic pharmaceutical products (i.e., monoclonal antibodies and recombinant proteins).[40] There are several issues that are unique to rAAV vectors. First, the relative abundances of VP1–VP3 in each capsid are thought to be stochastic,[41,42] and the resulting heterogeneity complicates analytical characterization of rAAV products. Low abundances of VP1, in particular, have been

Received 21 April 2021; accepted 16 August 2021;
https://doi.org/10.1016/j.omtm.2021.08.002.

Correspondence: Martin F. Jarrold, Chemistry Department, Indiana University, 800 East Kirkwood Avenue, Bloomington, IN 47405, USA.
E-mail: mfj@indiana.edu

© 2021 The Authors. This is an open access article under the CC BY license (http://creativecommons.org/licenses/by/4.0/).

Table 1. Promoter and genome for the AAV8 vectors studied in this work

Promoter and genome	No. of bases	Expected mass (MDa)[a]
Empty	0	0
CMV-CRE	2,219	0.683
CMV-GFP	2,544	0.783
CMV-mCherry	2,784	0.857
CAG-GFP	2,876	0.886
CAG-mCherry	2,895	0.892
EF1a-GFP	3,458	1.065
CBA-GFP	4,354	1.341
CMV-SaCas9	4,844	1.492

[a]From average of ionized + and – strands.

correlated with reduced potency.[43–45] Second, empty capsids are generated at high levels by current AAV vector generation systems.[46] Empty capsids can elevate the immune response and impair potency,[47,48] although there is also evidence that empty particles can act as decoys that mitigate immune response and macrophage clearance.[49,50] Regardless of whether empty particles are beneficial or detrimental, it is necessary to quantify their abundance. The same is true for capsids that have only packaged a partial genome.[28–30] The encapsidation of DNA impurities, such as heterogeneous fragments of host cellular, helper, or plasmid DNAs, can also occur.[51,52] The presence of both empty and partially packaged genomes adds additional heterogeneity, which further complicates analytical characterization of rAAV products. Finally, aggregation, which further increases the size, can reduce bioavailability and influence biodistribution, and it may also cause an immune response.[53]

Several methods have been used to determine the fraction of particles that are empty. These include optical density measurements where the ratio of the absorbance at 260 and 280 nm is used to estimate the fractions of empty and full particles.[46] Quantitative PCR (qPCR) and capsid ELISA (where the former determines the genome titer and the latter determines the total number of capsids) have also been widely used to determine the empty/full ratio.[54–56] The optical density approach, qPCR, and ELISA are sensitive to protein and nucleic acid impurities. Ion exchange chromatography, which has been widely used in the purification of rAAV vectors, has also been used to determine the empty/full ratio.[57,58] Negative stain transmission electron microscopy (TEM) is routinely used for empty/full quantification but tends to be imprecise. Cryogenic electron microscopy (cryo-EM) is better in this regard, but it is time-consuming and requires specialized instrumentation.[59] Finally, analytical ultracentrifugation (AUC) can provide empty/full quantification and information on capsids with a partial genome.[60] However, AUC is time-consuming and requires relatively large sample volumes at high concentrations.

Mass spectrometry (MS) has been an important tool in the development of many small-molecule pharmaceuticals. However, for large and heterogeneous samples, the loss of charge state resolution in the measured m/z (mass-to-charge ratio) spectrum has become a critical stumbling block. Thus, the intrinsic heterogeneity of rAAV vectors has precluded the widespread use of conventional MS for the analysis of intact AAV vectors.[41] Charge detection MS (CDMS),[61–65] where the m/z and charge are directly measured for each ion, can overcome this limitation.[30] Recent advances have allowed for the full characterization of rAAV samples with high throughput and minimal sample consumption (< 20 μL) at relatively low titers (10^{10} viral particles [vp]/mL).

Here, we report studies of rAAV8 control vectors containing a variety of target genome lengths produced in insect cells using baculovirus expression. CDMS and gel electrophoresis are used to characterize the genome content. We find evidence that the particles have packaged a broad distribution of heterogeneous DNA in addition to the genome of interest (GOI). The peak in the distribution of heterogeneous DNA is close to the packaging capacity. The results point to the value of additional characterization tools for the DNA content of rAAV vectors.

RESULTS

AAV8 vectors were purchased from Virovek where they were prepared in insect cells using baculovirus expression. The promoters and genomes for each vector along with the total number of bases (including the [inverted terminal repeats ITRs]) are summarized in Table 1. Figure 1A shows a CDMS spectrum measured for empty AAV8. The main peak is centered on a mass of around 3.7 MDa, with a high mass tail that extends to beyond 4 MDa (see inset). The sequence masses of VP1–VP3 for AAV8 are 81,667, 66,692, and 59,805 Da, respectively. As noted above, the VP1–VP3 ratio for capsids derived from HEK293 cells is around 1:1:10.[9–12] Capsids derived from baculovirus expression in insect cell lines appear to be less heterogeneous.[66] However, the ratios are not well established, and we use the 1:1:10 ratio as a starting point here. With this ratio, the expected mass of the capsid is 3.732 MDa, which is close to the mass of the main peak in Figure 1A.

The individual capsids in a sample do not have the same VP1–VP3 ratio. The copy numbers in each capsid are expected to follow a multinomial distribution,[30] and the presence of three capsid proteins in different ratios leads to a heterogeneous mass distribution. The CDMS spectrum in Figure 1A was recorded with a trapping time of 100 ms, which is too short to allow for charge state resolution, and so the subcomponents due to different numbers of VP1–VP3 are not resolved. The orange line in the inset of Figure 1A shows the calculated peak shape considering the resolution and heterogeneity. We hypothesize that the high mass tail on the main peak in Figure 1A is due to small DNA fragments (see below).

Charge and mass are independent quantities in CDMS, and structural information can be obtained by correlating them. Large ions generated by electrospray are thought to be produced by the charge residue mechanism,[67] where a water droplet deposits its charge on

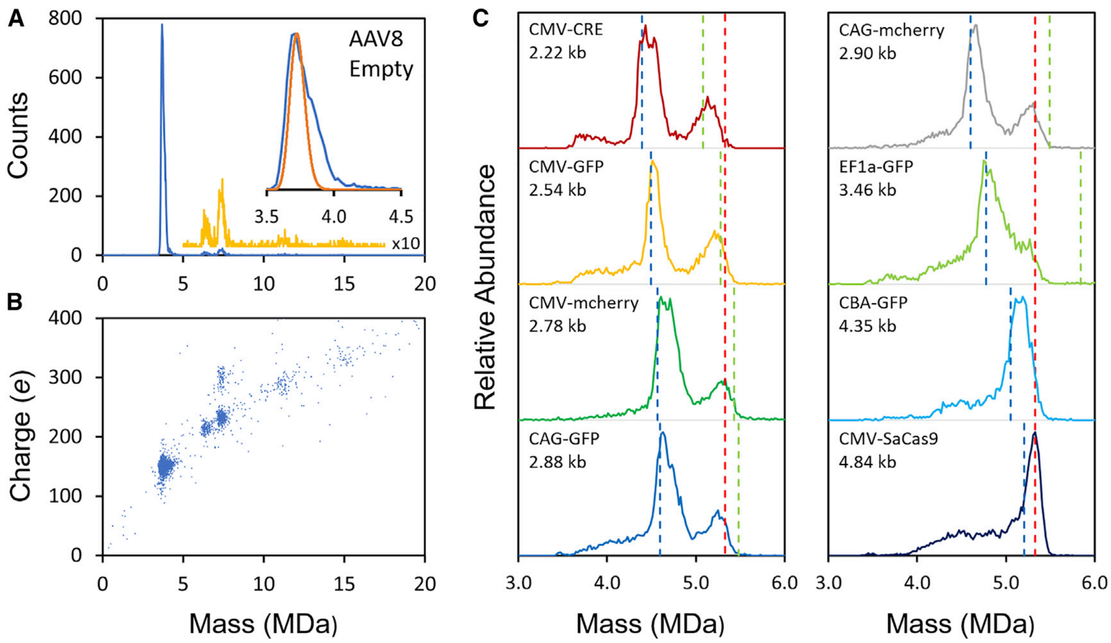

Figure 1. CDMS measurements for empty AAV8 and AAV8 with genomes

(A) Mass distribution for empty AAV8. The inset shows an expanded view of the main peak. The orange line shows the mass distribution calculated for a 1:1:10 ratio of VP1–VP3 considering the resolution for a trapping time of 100 ms. (B) Charge versus mass scatterplot for empty AAV8. (C) Mass distributions measured for AAV8 particles with the genomes in Table 1. The dashed blue lines show the expected masses for particles with a single genome, and the dashed green lines show the expected masses for two packaged genomes. The red dashed line at 5.33 MDa shows what appears to be the approximate packaging capacity of AAV8. In all cases, a bin width of 20 kDa was employed and all spectra contain around 10,000 ions.

the analyte as it evaporates away. Thus, the charge reflects an ion's size, and for a given mass, a higher charge indicates a larger or more extended structure.[68] Figure 1B shows a charge versus mass scatterplot for the mass distribution shown in Figure 1A. Each point in this plot represents a measurement for a single ion. The cluster of ions at around 3.7 MDa and 150 *e* (elementary charges) is due to the empty AAV8 capsid. There are several smaller clusters at higher mass and higher charge. The cluster at around 6.4 MDa (that comprises around 1.7% of the total) lacks a definitive assignment. It may be due to a peanut-like geometry where two partial capsids have fused. The clusters at around 7.4 MDa (~3.8%) are attributed to dimers of empty AAV8. A small cluster at around 11 MDa (~0.8%) is due to the trimer, and an even smaller cluster at around 15 MDa could be due to the tetramer. Note that the dimer at 7.4 MDa has two charge populations centered on around 225 and 300 *e*; these probably result from different dimer structures with the more highly charged cluster (around 20% of the dimer population) having a larger or more extended structure.

Figure 1C shows portions of the CDMS spectra measured for AAV8 particles with the genomes given in Table 1. The blue dashed lines in Figure 1C show the expected masses of AAV8 capsids that have packaged single genomes (calculated using the mass of the empty capsid from above and genome masses from Table 1). The CMV-CRE genome is small enough (2.22 kb) that two genomes could be packaged. The expected locations of the peaks due to AAV8 particles with two genomes are shown by the green dashed lines in Figure 1C. For CMV-CRE, there are peaks in the CDMS spectrum at masses that are slightly larger than the expected masses for capsids with one genome and two genomes. In both cases, the measured peaks are quite broad. The peak attributed to one genome is more abundant than the peak that could result from the packaging of two genomes.

The CDMS spectrum measured for CMV-GFP also shows two peaks. The peak at around 4.5 MDa is at a slightly higher mass than expected for a single genome (blue dashed line). However, two CMV-GFP genomes (5.09 kb) are larger than the wild-type genome (4.7 kb), and in this case, the second, higher-mass peak occurs at a mass that is slightly less than expected for two genomes (green dashed line).

The CDMS spectra for CMV-mCherry, CAG-GFP, and CAG-mCherry (Figure 1C) all show two peaks, similar to the spectrum for CMV-GFP discussed above. As the size of the genome increases, the peak attributed to a single full genome shifts to a higher mass, closely tracking the expected mass (blue dashed lines). While the theoretical masses for vectors that have packaged two complete genomes also shift to a higher mass, the experimental observations demonstrate that the second, higher-mass peaks in Figure 1C have a consistent mass, suggesting that the capsid packaging capacity is playing a role. The red dashed lines at 5.33 MDa in Figure 1C are guides indicating what appears to be the approximate packaging capacity of the AAV8 capsid.

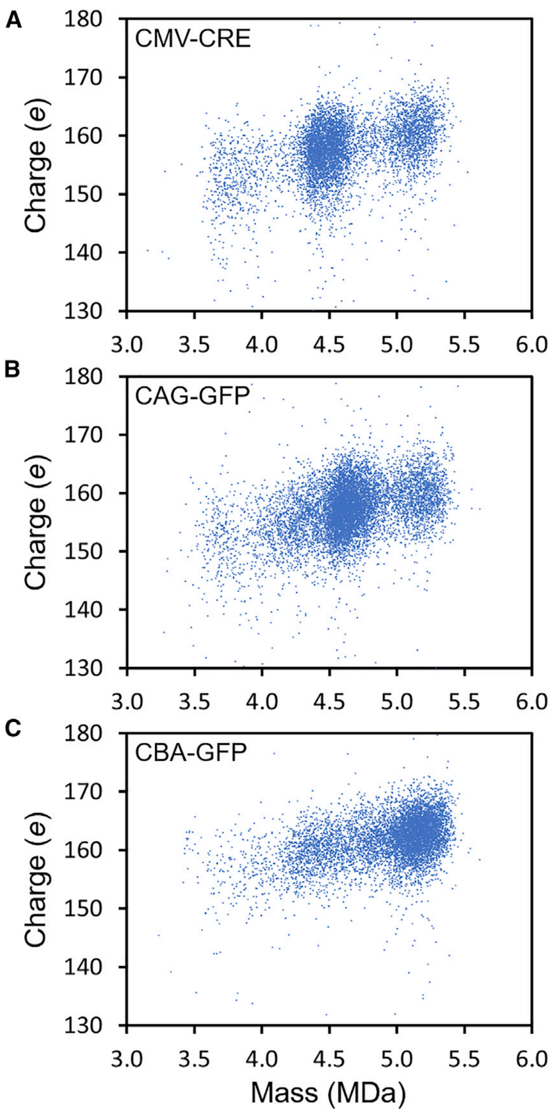

Figure 2. Charge versus mass scatterplots for representative genomes
(A) CMV-CRE. (B) CAG-GFP. (C) CBA-GFP.

The EF1a-GFP genome (3.46 kb) is significantly longer than CAG-mCherry (2.90 kb), and a second higher-mass peak is no longer resolved. Instead, a shoulder extends to beyond the red dashed line at 5.33 MDa. The genome for CBA-GFP (4.35 kb) is significantly longer than EF1a-GFP and the shoulder has now largely disappeared, apparently subsumed into the main peak. For CMV-SaCas9, the main peak is sharper and shifted to a slightly higher mass than expected for a single full genome. In addition to the main peak, there are significant low-mass tails for EF1a-GFP, CBA-GFP, and CMV-SaCas9. Similar low mass tails are evident in Figure 1C for all genomes. In some cases, the tail extends to the mass expected for the empty capsid, although there is not a defined peak at this mass. The intermediate masses (i.e., between the empty capsid and particles with a single full genome) probably result from packaging of a partial genome. Representative negative stain TEM images for some AAV8 samples are given in supplemental information where information about genome packaging deduced from the images is compared to the CDMS mass distributions.

The charge can provide structural information. We discussed an example above where the empty capsid dimer had two charge populations that were attributed to different geometries. For capsids where it appears that extra DNA is being packaged, the charge can provide information about the location of the DNA. If some of the DNA was outside the capsid the charge would be significantly larger than if the DNA were fully internalized. Figure 2 shows charge versus mass scatterplots for several representative genomes (CMV-CRE, CAG-GFP, and CBA-GFP). For CMV-CRE, the cluster of ions at around 4.5 MDa is assigned to particles that have packaged a single complete genome, and the cluster at around 5.2 MDa is assigned to particles that have packaged extra DNA, potentially two genomes. There is also a low-intensity, low-mass tail that extends to the mass of the empty particles (3.7 MDa). Despite the substantial mass increase from empty particles to particles with one full genome and then to particles with possibly two genomes, the charge increases only slightly. The increase is consistent with a small expansion of the capsid, suggesting that the DNA is fully internalized both when a single genome is packaged and when extra DNA, possibly two genomes, is packaged. A similar conclusion is reached from the CAG-GFP results in Figure 2. In this case, the cluster at around 4.7 MDa is due to the packaging of a single genome, and the cluster at around 5.2 MDa has packaged extra DNA. However, for CAG-GFP the high mass cluster cannot be due to the packaging of two complete genomes. There is also a significant cluster of ions at around 4.3 MDa that we attribute to particles with a partial genome. The clusters of ions due to packaging of a partial genome, a single genome, and the extra DNA all have similar charges, suggesting that the genomes are internalized. Finally, for CBA-GFP, the main cluster of ions at around 5.2 MDa is due to particles with a single genome and there is a long tail to lower mass attributable to particles with partial genomes. In this case, the genome is large enough that there is no evidence for the packaging of extra DNA. The charges for the main cluster of ions at around 5.2 MDa and for the low mass tail are comparable to the charges for CMV-CRE and CAG-GFP genomes and are consistent with a fully internalized genome.

The CDMS measurements for particles that have packaged smaller genomes show features that indicate that extra DNA is being packaged. There are at least two plausible explanations for the extra DNA. It could be due to the packaging of an additional GOI. In cases where packaging of two GOIs takes the particle beyond its capacity, the second GOI would be truncated.[16] Alternatively, it could be due to unknown DNA sequences that have been packaged up to the particle capacity and then possibly truncated. To distinguish between these two scenarios, we used gel electrophoresis to probe for single-stranded and double-stranded DNA (ssDNA and dsDNA).

Figure 3 shows the results of alkaline gel measurements that target ssDNA. For each sample, lanes with (+) and without (−) pretreatment

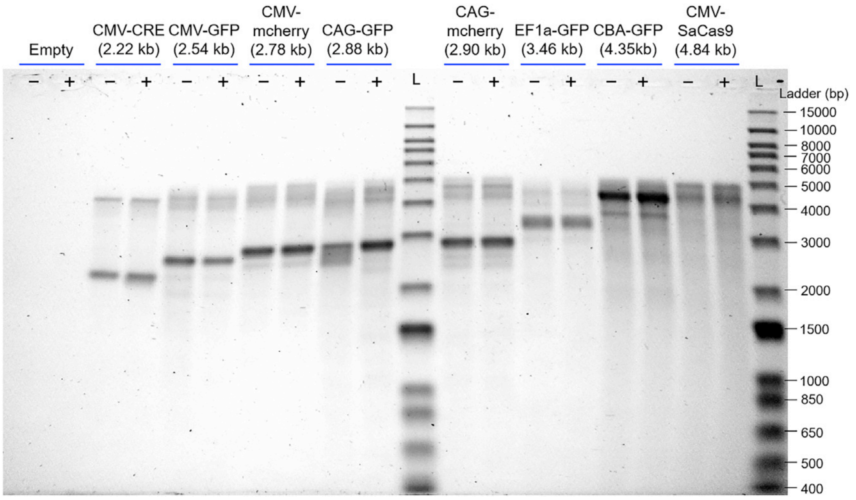

Figure 3. Alkaline gel measurements of ssDNA from AAV8 vectors

Two measurements are shown for each genome in Table 1: −, without DNase I; +, with DNase I; L, ladder. GOI lengths are shown in parentheses.

with DNase I are shown. Overall, the results have a lot in common with the CDMS spectra in Figure 1C. The main bands track the expected genome size, and the five smaller genomes (CMV-CRE, CMV-GFP, CMV-mCherry, CAG-GFP, and CAG-mCherry) that show a high mass peak in Figure 1C all have a second, lower-intensity band with a longer genome length in Figure 3. For some genomes there is a broad, low-intensity distribution at DNA lengths less than the GOI, which could correspond to intensity observed in Figure 1C between the masses of the empty capsid and particles with a single GOI. If the second higher-mass peak observed for CAG-mCherry in Figure 1C was due to the packaging of a full and a partial GOI, then a second band would be expected in Figure 3 at around 5.0 – 2.9 = 2.1 kb for the partial GOI, which is not observed. Similarly, there are no bands at the expected partial lengths of around 2.12 and 2.22 kb for CAG-GFP and CMV-mCherry, respectively. Thus, the results in Figure 3 appear to rule out the conjecture that the higher-mass peaks for the five smaller genomes result mainly from packaging a GOI plus a second whole or partial GOI. A densitometry analysis of the alkaline gels and a comparison to the CDMS intensities is provided in Supplemental information. The relative abundances of the higher-mass peaks/bands and the GOI peaks/bands are in pleasingly good agreement considering the drastically different nature of the measurements.

Figure 4 shows the results of TapeStation measurements that target dsDNA that result from base pairing in solution following disruption of the AAV capsids. AAV is expected to package both + (sense) and – (antisense) strands of DNA. The + and − strands hybridize to each other based on sequence complementarity when they are released from the capsid after heat denaturation. Three lanes are associated with each sample in Figure 4, corresponding to with (+) and without (−) DNase I pretreatment, and with DNase I pretreatment and purification (+*) (see Materials and methods). The main bands in Figure 4 track the expected genome size; however, note that there are no distinctive bands corresponding to lengths longer than the GOI for the five smallest genomes. Recall that bands were observed in the alkaline gels (that target ssDNA) for DNA lengths up to twice as long as the GOI. The absence of similar features here suggests that the longer lengths are largely heterogeneous species and do not base pair to a significant extent. In Figure 3, there are no prominent bands corresponding to lengths shorter than the GOI. There are, however, some bands corresponding to lengths shorter than the GOI in Figure 4. In particular, CAG-GFP, CAG-mCherry, and CBA-GFP show weaker intensity bands at less than the GOI. However, weak bands are also observed at less than the GOI for the longer genome lengths, such as CBA-GFP in Figure 4. The most likely explanation for these bands is that they are due to packaging of shorter DNA lengths (perhaps partial or truncated genome) that contributes to the intensity observed in Figure 1C between the masses of empty particles and particles with a single GOI.

Many of the peaks for the packaged particles (Figure 1C) are broader and shifted to a higher mass than expected. The peak for the empty capsid (Figure 1A) also has a significant high mass tail. We hypothesized that the excess mass could be due to small ssDNA fragments. We tried several approaches to remove the postulated impurities, including additional size-exclusion chromatography (SEC) cycles, dialysis using a variety of conditions, and heat incubation. Heat incubation provided the best results, leading to a significant narrowing of the peaks.

Figure 5 shows CDMS mass distribution recorded for EF1a-GFP before and after incubation at 65°C. The sample was incubated in a water bath for 15 min and then quenched in ice for 1 minute prior to solvent exchange into 200 mM ammonium acetate by SEC. After incubation, the peak due to the GOI narrows and shifts to a slightly lower mass. The black vertical line is a guide to help illustrate this behavior. The orange line in Figure 5 shows the expected peak width, considering capsid heterogeneity and instrumental resolution. A narrowing of the peak and a shift to a slightly lower mass occurred for the empty particles and for most of the other genomes listed in Table 1. A number of previous studies have reported on temperature-induced changes in AAV vectors.[69–72] A more detailed accounting of the CDMS measurements will be reported elsewhere. We mention these results here because we use the masses from the heat-treated samples below.

Upon incubation, the center mass of the empty capsid shifted from 3.710 to 3.678 MDa. The true mass is probably slightly less than this value because of counterions and residual salt, which are known

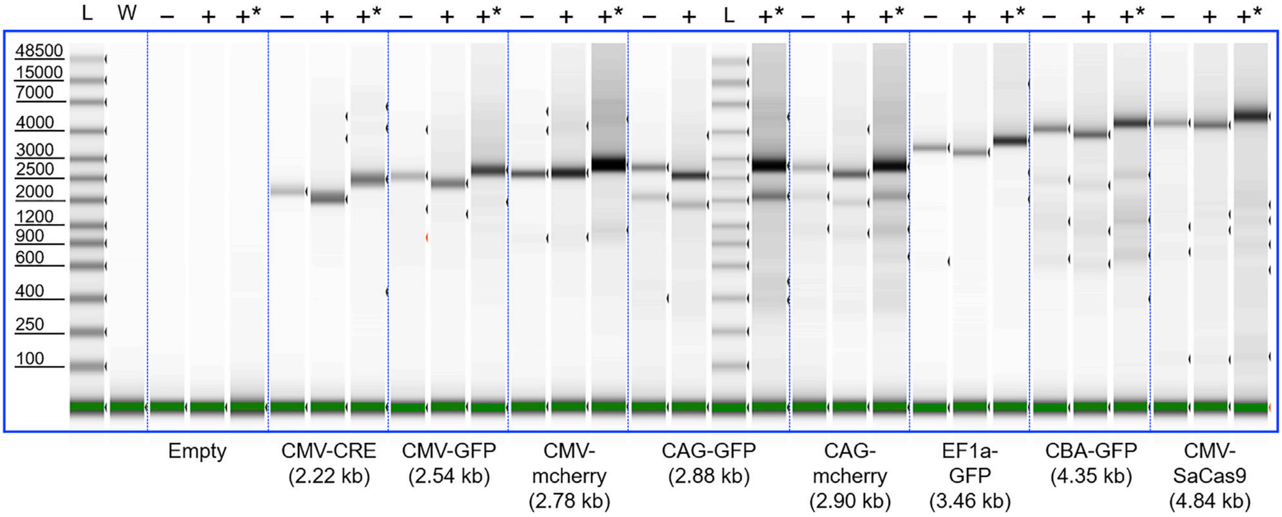

Figure 4. TapeStation measurements of dsDNA from AAV8 vectors

Three measurements are shown for each genome in Table 1: –, without DNase I; +, with DNase I; +*, with DNase I and purified (see Materials and methods for details). L, ladder; W, water. The green band at the bottom of the gel indicates the internal marker at 100 bp. GOI lengths are shown in parentheses.

to increase the mass slightly (around 0.3%–0.5%) under the native electrospray conditions used here. The sequence mass for a 1:1:10 ratio of VP1–VP3 is 3.732 MDa. Note, however, that post-translational modifications[73] (including truncation of VP1 and VP2)[74] could affect the mass. Thus, a measured mass slightly less than the sequence mass could be explained by N-terminal processing or by lower levels of VP1 and/or VP2 than the expected 1:1:10 ratio. Vectors derived from recombinant baculovirus using insect cells lines have been found to incorporate lower levels of VP1 than capsids prepared in mammalian cell lines.[66]

The mass of the genome can be deduced from the difference between the measured masses of the empty and full capsids. The resulting genome masses are plotted against the calculated sequence masses (Table 1) as the red points in Figure 6. We used the masses determined after incubation. The dashed black line in Figure 6 is a guide showing the expected behavior when the measured genome mass equals the sequence mass. The measured genome masses are 1%–3% larger than sequence masses. The solid red line is a least squared fit to the points. The line has an intercept of −0.016 MDa and a slope of 1.041. The excess mass may be due, at least partly, to counterions associated with the ssDNA. The calculated sequence masses used in Figure 6 are for DNA with ionized backbone phosphate groups, and there maybe counterions associated with them. It is also common in ssDNA and ssRNA viruses for basic sites inside the capsid to neutralize some of the charge.[75,76]

To better understand the results in Figure 6, consider a situation where the internal surface of the capsid has i basic sites. In the empty capsid there are probably counterions associated with each basic site. The average mass of these counter ions is m^-. Assume the genome has j nucleotides and the average mass of each ionized nucleotide is m_N. There is a counterion associated with each nucleotide that is not neutralized by the basic sites on the inside of the capsid. The average mass of these counter ions is m^+. If the average mass of the empty capsid without counter ions is M, then the average mass of the empty capsid with internal counter ions is

$$M_E = M + (i \times m^-) \tag{1}$$

and the average mass of the full capsid is

$$M_F = M + (j \times m_N) + [(j - i) \times m^+]. \tag{2}$$

The difference between the mass of the full and empty particles is

$$M_F - M_E = \left[\left(1 + \frac{m^+}{m_N}\right) \times (j \times m_N)\right] - [i \times (m^+ + m^-)]. \tag{3}$$

According to this model, a plot of the difference between the full and empty particles ($M_F - M_E$) against the genome mass ($j \times m_N$) has a slope of $(1 + (m^+ / m_N))$ and an intercept of $-(i \times [m^+ + m^-])$. Thus, the slope reflects the mass of the counterions associated with the DNA and the intercept reflects the number of basic sites on the inside of the capsid.

The slope is expected to be 1.075 if all of the counter ions are Na^+, 1.059 for NH_4^+, 1.039 for Mg^{2+}, and 1.003 for H^+ (i.e., unionized). While it is not possible to draw a conclusion about the nature of the counterions, the measured slope (1.041) is in the expected range. The small negative intercept (0.016 MDa) indicates that the number of excess basic sites per capsid protein is small (~3), which is consistent with the known structure of the AAV8 capsid.[77] These results support the idea that counterions are mainly responsible for the small

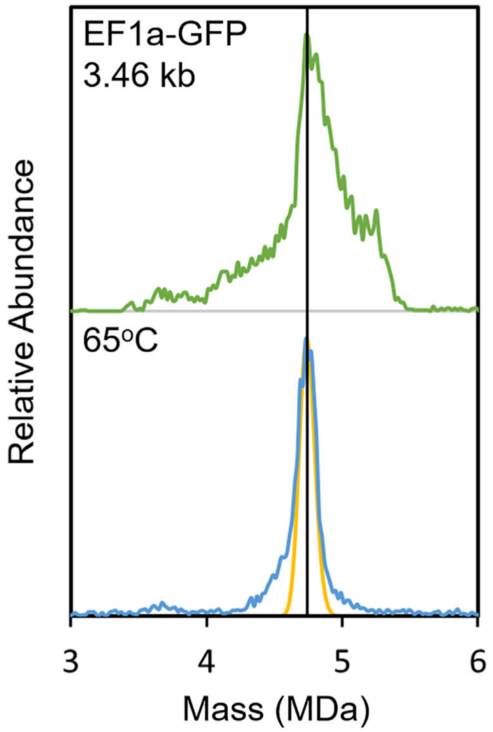

Figure 5. Incubation of AAV8 particles with EF1a-GFP genome
The CDMS mass distribution measured after incubation at 65°C for 15 min is shown in blue. The distribution measured before incubation is shown in green for comparison. The orange line shows the expected peak width considering the capsid heterogeneity and instrumental resolution.

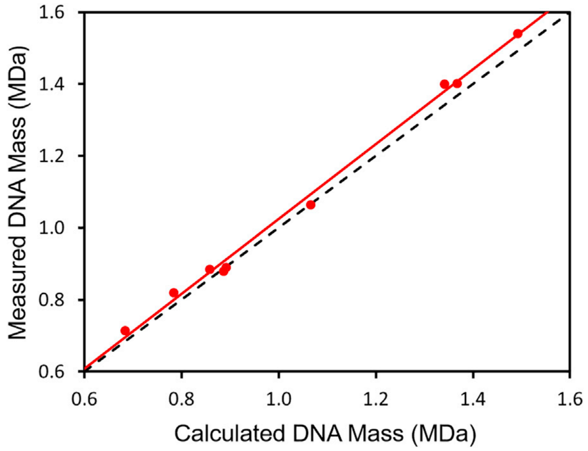

Figure 6. The measured DNA masses plotted against the DNA sequence masses
The measured masses were obtained from the difference between the masses measured for the full and empty particles and the sequence masses are from Table 1. The red points are the experimental data. The dashed black line shows the expected behavior when the measured DNA mass equals the expected mass (slope = 1.0). The red line shows a least-squares fit to the data points. The slope is 1.041, the intercept is −0.016 MDa, and R^2 is 0.9956.

difference between the measured DNA masses and the expected masses.

Because the unincubated Virovek samples were so heterogeneous, we were interested in comparing the results to a sample that had been more extensively purified. Figure 7 shows CDMS results for the ATCC (American Type Culture Collection) AAV8 reference standard.[78,79] The blue line in Figure 7A shows the measured mass distribution. The spectrum is dominated by a peak at close to the mass expected for AAV8 particles that have packaged a full genome. The dashed red line shows the expected mass distribution calculated considering the expected capsid heterogeneity and instrument resolution. The width of the measured peak is in good agreement with the expected peak width. The yellow line in Figure 7 shows the counts ×10 and offset vertically. Only a few ions were detected with masses in the range expected for empty AAV8 (~3.7 MDa), reflecting expectations from the closely matched capsid titer (5.50×10^{11} particles/mL) and the genome titer (5.75×10^{11} vg/mL) measured by the AAV8 working group.[75] There is, however, a significant number of ions (around 14% of the total) with masses between the empty and full particles. These ions are presumably due to capsids that contain less than the full genome. There appear to be two components that must contain different amounts of DNA: a broad peak centered around 4.4 MDa and a shoulder at around 4.9 MDa. Because there is not a significant peak for the empty particle in Figure 7A, it is not possible to accurately compare the difference between the masses of the empty and full particles to the genome mass. However, we did observe that temperature cycling caused the main peak to shift to a lower mass by up to 40 kDa. This suggests that the capsids contain small lengths of extraneous DNA that is co-packaged with the GOI. Figure 7B shows the charge versus mass scatterplot for the AAV8 reference standard. This plot should be compared to the results in Figure 1B where there was evidence for several different types of higher-order multimers. No multimers were detected for the reference standard.

DISCUSSION

Quantification of empty/full ratios is the most basic AAV vector quality metric. From extensive studies with CDMS, this technology appears to be universally applicable to all AAV serotypes measured to date, including all natural AAV serotypes and many non-natural ones, and it provides an accurate and precise measurement of the empty/full ratio. In this work we show that CDMS can go beyond simple empty/full quantification of AAV vectors to provide information on the mass distribution of the packaged DNA as well as information about multimer populations. In addition, the charge provides orthogonal information about the structure, which in the present case shows that all of the DNA is internalized rather than being attached to the outside of the capsid.

All of the AAV vectors studied herein have a prominent peak at close to the mass expected for particles with a single GOI. For vectors where the length of the GOI is short compared to the length of the WT

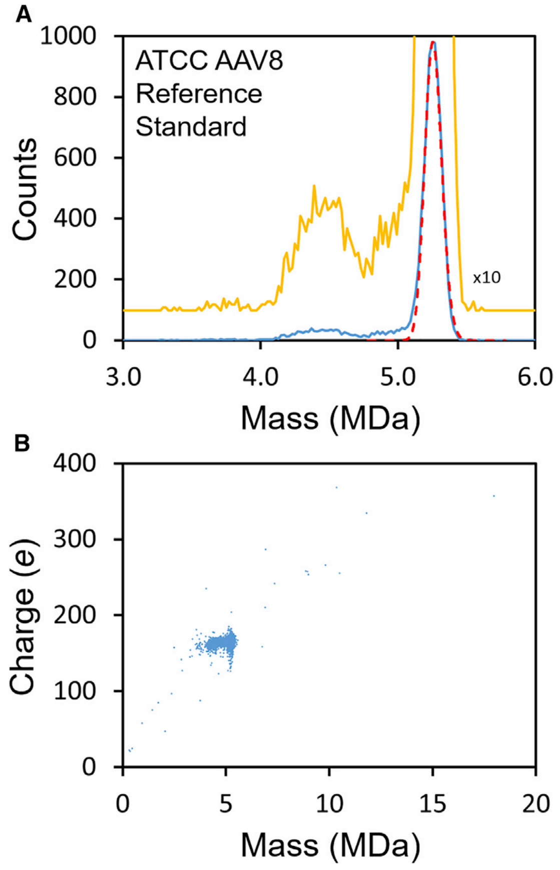

Figure 7. CDMS measurements for ATCC AAV8 reference standard

(A) plot of the mass distribution (blue line). The yellow line shows the spectrum ×10 and offset vertically. The dashed red line shows the distribution expected when the capsid heterogeneity and experimental resolution are included. (B) charge versus mass scatterplot where the measurement for each ion is represented by a point.

genome (4.7 kb) there is also a peak at a mass close to the packaging capacity of the AAV capsid. For CMV-CRE (2.22 kb) the genome is short enough that two copies could be packaged. However, for CMV-GFP (2.54 kb), CMV-mCherry (2.78 kb), CAG-GFP (2.88 kb), and CAG-mCherry (2.90 kb) the genome is too long to package two complete copies. For these GOIs, the second peak could be due to packaging one full genome and one partial genome, a possibility raised by Frizzell and colleagues[16] some time ago. However, gel electrophoresis measurements indicate that the peak near the packaging capacity results mainly from a single strand of heterogeneous DNA that is presumably plasmid derived or from the host cells. That the distribution for heterogeneous DNA peaks at close to the packaging capacity is consistent with headful packaging.

For unincubated samples the width of the peak attributed to the GOI is broader than the width of the peak due to the empty capsid. Temperature cycling the AAV samples sharpens the peaks in the CDMS mass distribution and causes them to shift to a slightly lower mass. These changes lead to measured masses in better agreement with expectations for the targeted GOI. After temperature cycling the measured genome masses (obtained by subtracting the measured mass of the empty particle from the measured mass of the particle with a genome) are 1%–3% larger than the sequence masses. This small difference can be accounted for by counterions packaged with the DNA. An underlying assumption of this analysis is that the capsid masses for the empty and full particles are identical or at least very similar (i.e., the relative amounts of VP1–VP3 are very similar for both the empty and full particles). This appears to be a good assumption, at least for the samples examined in the present study.

High-mass ions derived from solutions of electrospray-compatible salt solutions (such as ammonium acetate employed here) are known to have measured masses that are a fraction of a percent larger than the expected mass. The excess mass is attributed to counterions and residual solvent. The changes in the mass distributions on temperature cycling have a different origin because all samples were solvent exchanged into the same electrospray-compatible solution (ammonium acetate) after temperature cycling. We suspect that the high mass tail on the peak for the empty particles and the broad GOI peak widths are due to small DNA fragments from the host cells that are co-packaged along with the target genome. By temperature cycling, it is possible to remove some, but apparently not all, of the DNA fragments: we could not find conditions where the high mass tail for the empty particles was completely removed.

Given the heterogeneous nature of the DNA packaged into the capsids, a measurement focused exclusively on the empty/full ratio is insufficient to characterize the broad distribution of DNA incorporated into the particles studied here. Proper understanding of the packaged genome can be obtained by using additional techniques shown herein, such as CDMS, TapeStation, and gel electrophoresis, or alternative technologies that include next-generation sequencing.

MATERIALS AND METHODS

AAV samples

The AAV8 vectors and empty AAV8 were purchased from Virovek where they were prepared using baculovirus expression in Sf9 insect cells. Empty AAV8 and the AAV8 vectors were purified by the manufacturer using ultracentrifugation with a CsCl gradient. The AAV8 reference standard material[74,75] AAV8RSM (VR-1816) was purchased from ATCC. All samples were stored at −80°C. Stored aliquots were thawed at room temperature, and prior to electrospray 10- to 20-μL volumes were exchanged into 200 mM ammonium acetate (Honeywell 631-31-8) solution at pH 7.5 using Micro Bio-Spin columns (Bio-Rad, 7326221).

CDMS

CDMS allows mass distribution to be measured for heterogeneous and high molecular weight samples that are not accessible by conventional MS. In CDMS, the masses of individual ions are determined from simultaneous measurements of each ion's *m*/*z* ratio and charge. Mass measurements are performed for thousands of ions and then the results are binned to yield a mass distribution. The homebuilt CDMS

instrument employed here has been described previously.[80–82] Samples were electrosprayed using a commercial nanoelectrospray source (Advion TriVersa NanoMate), and the resulting ions enter the instrument though a metal capillary. The ions pass through several stages of differential pumping to separate them from the ambient gas that enters the capillary with the ions. They are then accelerated to a nominal energy of 100 eV/z and focused into a dual hemispherical deflection energy analyzer (HDA) that transmits a narrow band of ion energies centered on the nominal ion energy. Ions that pass through the HDA are focused into an electrostatic linear ion trap (ELIT), where trapped ions oscillate back and forth through a metal detection cylinder. When an ion enters the cylinder, it induces a charge that is detected by a charge sensitive preamplifier. The resulting signal is amplified, digitized, and analyzed using fast Fourier transforms.[78] The oscillation frequency and magnitudes are related to the ion's m/z and charge, respectively. Multiple ion events (where two or more ions are trapped simultaneously) and events where ions were not trapped for the full trapping period were discarded. The uncertainty in the charge measurement scales with (trapping time)$^{-1/2}$ and, with the 100 ms trapping time employed here, the uncertainty (RMSD) is around 1 e. Note that this uncertainty is, to the first order, independent of the charge. Most CDMS spectra reported herein contain around 5,000 ions and took 20–40 min to measure.

ssDNA measured by alkaline gel

Two samples of each genome containing approximately 2.4×10^{11} copies of AAV vector genome were aliquoted. One sample was treated with DNase I (New England Biolabs) at 37°C. After 60 min, 5 mM EDTA (Sigma) was added to quench the reaction. 6× alkaline loading buffer (6 mg/mL xylene cyanol, 300 mM NaOH, 6 mM EDTA, 0.18 g/mL Ficoll) was then added to both samples, followed by heat treatment at 80°C for 10 min. The samples were then kept on ice until they were ready to be loaded onto the gel. 20 μL of the denatured samples was loaded onto 1% alkaline agarose gel (1% agarose, 50 mM NaOH, 1 mM EDTA). The denatured DNA was separated at 22 V, for 18 h at 2°C–8°C. The gel was agitated in 1× neutralization buffer (0.5 M Tris-HCl [pH 7.5], 1 M NaCl) for 30 min, and then this procedure was repeated. The neutralized gel was stained with SYBR Gold (Invitrogen) in 1× TAE (Tris-acetate-EDTA) buffer (Bio-Rad) for 60 min and imaged using fluorescence mode with a blue filter (Bio-Rad Gel EZ Doc). An Invitrogen 1-kb Plus DNA Ladder (PN 10787026) was used for calibration.

dsDNA measured by an Agilent Technologies TapeStation

Three samples of each genome containing approximately 4×10^{11} copies of AAV vector genome were aliquoted. Two of the samples were treated with DNase I (New England Biolabs) at 37°C. After 60 min, 5 mM EDTA (Sigma) was added to both samples to quench the reaction. All three samples (DNase I treated and untreated) were then heated to 80°C for 10 min. Conversion of ssDNA to dsDNA was performed by transferring the samples to a water bath at 65°C, and then the water bath was turned off so that the temperature dropped to 40°C–43°C during the course of 60 min. The DNA in one of the samples treated with DNase I was purified using a QIAquick PCR purification kit (QIAGEN). 2-μL samples of vector DNA and 10 μL of sample buffer (Agilent) were vortexed at 2,200 rpm for 1 min. Genomic DNA ScreenTape (Agilent, PN 5067-5365) and genomic DNA reagents (Agilent, PN 5067-5366) were used to measure the size of the dsDNA using an Agilent 4200 TapeStation.

Negative-stain TEM

TEM measurements were performed at Indiana University's Electron Microscopy Center on a JEOL JEM 1010 microscope. EM samples were prepared on Formvar/carbon film copper grids that were glow discharged with a PELCO easiGlow glow discharger. 10 μL of sample was dried for 10 min before excess solvent was removed by blotting. Negative staining was done with 10 μL of 2% uranyl acetate with immediate removal. Images were collected with ×50,000 magnification.

SUPPLEMENTAL INFORMATION

Supplemental information can be found online at https://doi.org/10.1016/j.omtm.2021.08.002.

ACKNOWLEDGMENTS

The research described herein was supported by a grant from Pfizer to Indiana University. We are grateful to Lohra Miller for help with the TEM measurements.

AUTHOR CONTRIBUTIONS

T.W.P. and M.F.J. conceived the study. L.F.B. and B.E.D. performed the CDMS measurements. Y.-T.C. performed the gel electrophoresis measurements. All authors contributed to the interpretation of the results. L.F.B. and M.F.J. wrote the first draft of the manuscript. All authors edited the manuscript.

DECLARATION OF INTERESTS

B.E.D. and M.F.J. are shareholders in Megadalton Solutions, a company that is engaged in commercializing CDMS. The remaining authors declare no competing interests.

REFERENCES

1. Atchison, R.W., Casto, B.C., and Hammon, W.M. (1966). Electron microscopy of adenovirus-associated virus (AAV) in cell cultures. Virology *29*, 353–357.
2. Hoggan, M.D., Blacklow, N.R., and Rowe, W.P. (1966). Studies of small DNA viruses found in various adenovirus preparations: Physical, biological, and immunological characteristics. Proc. Natl. Acad. Sci. USA *55*, 1467–1474.
3. Srivastava, A., Lusby, E.W., and Berns, K.I. (1983). Nucleotide sequence and organization of the adeno-associated virus 2 genome. J. Virol. *45*, 555–564.
4. Meier, A.F., Fraefel, C., and Seyffert, M. (2020). The interplay between adeno-associated virus and its helper viruses. Viruses *12*, 662.
5. Samulski, R.J., Berns, K.I., Tan, M., and Muzyczka, N. (1982). Cloning of adeno-associated virus into pBR322: Rescue of intact virus from the recombinant plasmid in human cells. Proc. Natl. Acad. Sci. USA *79*, 2077–2081.
6. Samulski, R.J., Srivastava, A., Berns, K.I., and Muzyczka, N. (1983). Rescue of adeno-associated virus from recombinant plasmids: Gene correction within the terminal repeats of AAV. Cell *33*, 135–143.
7. Wang, D., Tai, P.W.L., and Gao, G. (2019). Adeno-associated virus vector as a platform for gene therapy delivery. Nat. Rev. Drug Discov. *18*, 358–378.

8. Keeler, A.M., and Flotte, T.R. (2019). Recombinant adeno-associated virus gene therapy in light of Luxturna (and Zolgensma and Glybera): Where are we, and how did we get here? Annu. Rev. Virol. *6*, 601–621.

9. Rose, J.A., Maizel, J.V., Jr., Inman, J.K., and Shatkin, A.J. (1971). Structural proteins of adenovirus-associated viruses. J. Virol. *8*, 766–770.

10. Johnson, F.B., Ozer, H.L., and Hoggan, M.D. (1971). Structural proteins of adenovirus-associated virus type 3. J. Virol. *8*, 860–863.

11. Buller, R.M.L., and Rose, J.A. (1978). Characterization of adenovirus-associated virus-induced polypeptides in KB cells. J. Virol. *25*, 331–338.

12. Samulski, R.J., and Muzyczka, N. (2014). AAV-Mediated gene therapy for research and therapeutic purposes. Annu. Rev. Virol. *1*, 427–451.

13. Kronenberg, S., Kleinschmidt, J.A., and Böttcher, B. (2001). Electron cryo-microscopy and image reconstruction of adeno-associated virus type 2 empty capsids. EMBO Rep. *2*, 997–1002.

14. Kaludov, N., Padron, E., Govindasamy, L., McKenna, R., Chiorini, J.A., and Agbandje-McKenna, M. (2003). Production, purification and preliminary X-ray crystallographic studies of adeno-associated virus serotype 4. Virology *306*, 1–6.

15. Nam, H.J., Lane, M.D., Padron, E., Gurda, B., McKenna, R., Kohlbrenner, E., Aslanidi, G., Byrne, B., Muzyczka, N., Zolotukhin, S., and Agbandje-McKenna, M. (2007). Structure of adeno-associated virus serotype 8, a gene therapy vector. J. Virol. *81*, 12260–12271.

16. Dong, J.Y., Fan, P.D., and Frizzell, R.A. (1996). Quantitative analysis of the packaging capacity of recombinant adeno-associated virus. Hum. Gene Ther. *7*, 2101–2112.

17. Hermonat, P.L., Quirk, J.G., Bishop, B.M., and Han, L. (1997). The packaging capacity of adeno-associated virus (AAV) and the potential for wild-type-plus AAV gene therapy vectors. FEBS Lett. *407*, 78–84.

18. Zhou, X., and Muzyczka, N. (1998). In vitro packaging of adeno-associated virus DNA. J. Virol. *72*, 3241–3247.

19. Allocca, M., Doria, M., Petrillo, M., Colella, P., Garcia-Hoyos, M., Gibbs, D., Kim, S.R., Maguire, A., Rex, T.S., Di Vicino, U., et al. (2008). Serotype-dependent packaging of large genes in adeno-associated viral vectors results in effective gene delivery in mice. J. Clin. Invest. *118*, 1955–1964.

20. Hirsch, M.L., Agbandje-McKenna, M., and Samulski, R.J. (2010). Little vector, big gene transduction: Fragmented genome reassembly of adeno-associated virus. Mol. Ther. *18*, 6–8.

21. Lai, Y., Yue, Y., and Duan, D. (2010). Evidence for the failure of adeno-associated virus serotype 5 to package a viral genome > or = 8.2 kb. Mol. Ther. *18*, 75–79.

22. Wu, Z., Yang, H., and Colosi, P. (2010). Effect of genome size on AAV vector packaging. Mol. Ther. *18*, 80–86.

23. Dong, B., Nakai, H., and Xiao, W. (2010). Characterization of genome integrity for oversized recombinant AAV vector. Mol. Ther. *18*, 87–92.

24. Berns, K.I., and Rose, J.A. (1970). Evidence for a single-stranded adenovirus-associated virus genome: Isolation and separation of complementary single strands. J. Virol. *5*, 693–699.

25. Ling, C., Wang, Y., Lu, Y., Wang, L., Jayandharan, G.R., Aslanidi, G.V., Li, B., Cheng, B., Ma, W., Lentz, T., et al. (2015). The adeno-associated virus genome packaging puzzle. J. Mol. Genet. Med. *9*, 1000175.

26. Myers, M.W., and Carter, B.J. (1980). Assembly of adeno-associated virus. Virology *102*, 71–82.

27. Bleker, S., Sonntag, F., and Kleinschmidt, J.A. (2005). Mutational analysis of narrow pores at the fivefold symmetry axes of adeno-associated virus type 2 capsids reveals a dual role in genome packaging and activation of phospholipase A2 activity. J. Virol. *79*, 2528–2540.

28. Hauswirth, W.W., and Berns, K.I. (1979). Adeno-associated virus DNA replication: Nonunit-length molecules. Virology *93*, 57–68.

29. Laughlin, C.A., Myers, M.W., Risin, D.L., and Carter, B.J. (1979). Defective-interfering particles of the human parvovirus adeno-associated virus. Virology *94*, 162–174.

30. Pierson, E.E., Keifer, D.Z., Asokan, A., and Jarrold, M.F. (2016). Resolving adeno-associated viral particle diversity with charge detection mass spectrometry. Anal. Chem. *88*, 6718–6725.

31. Chamberlain, K., Riyad, J.M., and Weber, T. (2016). Expressing transgenes that exceed the packaging capacity of adeno-associated virus capsids. Hum. Gene Ther. Methods *27*, 1–12.

32. Hirsch, M.L., Wolf, S.J., and Samulski, R.J. (2016). Delivering transgenic DNA exceeding the carrying capacity of AAV vectors. Methods Mol. Biol *1382*, 21–39.

33. Wright, J.F. (2008). Manufacturing and characterizing AAV-based vectors for use in clinical studies. Gene Ther. *15*, 840–848.

34. Clément, N., and Grieger, J.C. (2016). Manufacturing of recombinant adeno-associated viral vectors for clinical trials. Mol. Ther. Methods Clin. Dev. *3*, 16002.

35. Urabe, M., Ding, C., and Kotin, R.M. (2002). Insect cells as a factory to produce adeno-associated virus type 2 vectors. Hum. Gene Ther. *13*, 1935–1943.

36. Chen, H. (2008). Intron splicing-mediated expression of AAV Rep and Cap genes and production of AAV vectors in insect cells. Mol. Ther. *16*, 924–930.

37. Virag, T., Cecchini, S., and Kotin, R.M. (2009). Producing recombinant adeno-associated virus in foster cells: Overcoming production limitations using a baculovirus-insect cell expression strategy. Hum. Gene Ther. *20*, 807–817.

38. Kurasawa, J.H., Park, A., Sowers, C.R., Halpin, R.A., Tovchigrechko, A., Dobson, C.L., Schmelzer, A.E., Gao, C., Wilson, S.D., and Ikeda, Y. (2020). Chemically defined, high-density insect cell-based expression system for scalable AAV vector production. Mol. Ther. Methods Clin. Dev. *19*, 330–340.

39. Rumachik, N.G., Malaker, S.A., Poweleit, N., Maynard, L.H., Adams, C.M., Leib, R.D., Cirolia, G., Thomas, D., Stamnes, S., Holt, K., et al. (2020). Methods matter: Standard production platforms for recombinant AAV produce chemically and functionally distinct vectors. Mol. Ther. Methods Clin. Dev. *18*, 98–118.

40. Gimpel, A.L., Katsikis, G., Sha, S., Maloney, A.J., Hong, M.S., Nguyen, T.N.T., Wolfrum, J., Springs, S.L., Sinskey, A.J., Manalis, S.R., et al. (2021). Analytical methods for process and product characterization of recombinant adeno-associated virus-based gene therapies. Mol. Ther. Methods Clin. Dev. *20*, 740–754.

41. Snijder, J., van de Waterbeemd, M., Damoc, E., Denisov, E., Grinfeld, D., Bennett, A., Agbandje-McKenna, M., Makarov, A., and Heck, A.J.R. (2014). Defining the stoichiometry and cargo load of viral and bacterial nanoparticles by Orbitrap mass spectrometry. J. Am. Chem. Soc. *136*, 7295–7299.

42. Wörner, T.P., Snijder, J., Bennett, A., Agbandje-McKenna, M., Makarov, A.A., and Heck, A.J.R. (2020). Resolving heterogeneous macromolecular assemblies by Orbitrap-based single-particle charge detection mass spectrometry. Nat. Methods *17*, 395–398.

43. Grieger, J.C., Johnson, J.S., Gurda-Whitaker, B., Agbandje-McKenna, M., and Samulski, R.J. (2007). Surface-exposed adeno-associated virus Vp1-NLS capsid fusion protein rescues infectivity of noninfectious wild-type Vp2/Vp3 and Vp3-only capsids but not that of fivefold pore mutant virions. J. Virol. *81*, 7833–7843.

44. Popa-Wagner, R., Porwal, M., Kann, M., Reuss, M., Weimer, M., Florin, L., and Kleinschmidt, J.A. (2012). Impact of VP1-specific protein sequence motifs on adeno-associated virus type 2 intracellular trafficking and nuclear entry. J. Virol. *86*, 9163–9174.

45. Bosma, B., du Plessis, F., Ehlert, E., Nijmeijer, B., de Haan, M., Petry, H., and Lubelski, J. (2018). Optimization of viral protein ratios for production of rAAV serotype 5 in the baculovirus system. Gene Ther. *25*, 415–424.

46. Sommer, J.M., Smith, P.H., Parthasarathy, S., Isaacs, J., Vijay, S., Kieran, J., Powell, S.K., McClelland, A., and Wright, J.F. (2003). Quantification of adeno-associated virus particles and empty capsids by optical density measurement. Mol. Ther. *7*, 122–128.

47. Mingozzi, F., and High, K.A. (2011). Immune responses to AAV in clinical trials. Curr. Gene Ther. *11*, 321–330.

48. Gao, K., Li, M., Zhong, L., Su, Q., Li, J., Li, S., He, R., Zhang, Y., Hendricks, G., Wang, J., and Gao, G. (2014). Empty virions in AAV8 vector preparations reduce transduction efficiency and may cause total viral particle dose-limiting side-effects. Mol. Ther. Methods Clin. Dev. *1*, 20139.

49. Mingozzi, F., Anguela, X.M., Pavani, G., Chen, Y., Davidson, R.J., Hui, D.J., Yazicioglu, M., Elkouby, L., Hinderer, C.J., Faella, A., et al. (2013). Overcoming preexisting humoral immunity to AAV using capsid decoys. Sci. Transl. Med. *5*, 194ra92.

50. Aalbers, C.J., Broekstra, N., van Geldorp, M., Kramer, E., Ramiro, S., Tak, P.P., Vervoordeldonk, M.J., and Finn, J.D. (2017). Empty capsids and macrophage

inhibition/depletion increase rAAV transgene expression in joints of both healthy and arthritic mice. Hum. Gene Ther. *28*, 168–178.

51. Smith, P.H., Wright, J.F., Qu, G., Patarroyo-White, S., Parker, A., and Sommer, J.M. (2003). Packaging of host cell and plasmid DNA into recombinant adeno-associated virus particles produced by triple transfection. Mol. Ther. *7* (*Suppl*), S348.
52. Chadeuf, G., Ciron, C., Moullier, P., and Salvetti, A. (2005). Evidence for encapsidation of prokaryotic sequences during recombinant adeno-associated virus production and their in vivo persistence after vector delivery. Mol. Ther. *12*, 744–753.
53. Wright, J.F., Le, T., Prado, J., Bahr-Davidson, J., Smith, P.H., Zhen, Z., Sommer, J.M., Pierce, G.F., and Qu, G. (2005). Identification of factors that contribute to recombinant AAV2 particle aggregation and methods to prevent its occurrence during vector purification and formulation. Mol. Ther. *12*, 171–178.
54. Kuck, D., Kern, A., and Kleinschmidt, J.A. (2007). Development of AAV serotype-specific ELISAs using novel monoclonal antibodies. J. Virol. Methods *140*, 17–24.
55. Wang, F., Cui, X., Wang, M., Xiao, W., and Xu, R. (2013). A reliable and feasible qPCR strategy for titrating AAV vectors. Med. Sci. Monit. Basic Res. *19*, 187–193.
56. Wang, Y., Menon, N., Shen, S., Feschenko, M., and Bergelson, S. (2020). A qPCR method for AAV genome titer with ddPCR-level of accuracy and precision. Mol. Ther. Methods Clin. Dev. *19*, 341–346.
57. Lock, M., Alvira, M.R., and Wilson, J.M. (2012). Analysis of particle content of recombinant adeno-associated virus serotype 8 vectors by ion-exchange chromatography. Hum. Gene Ther. Methods *23*, 56–64.
58. Wang, C., Mulagapati, S.H.R., Chen, Z., Du, J., Zhao, X., Xi, G., Chen, L., Linke, T., Gao, C., Schmelzer, A.E., and Liu, D. (2019). Developing an anion exchange chromatography assay for determining empty and full capsid contents in AAV6.2. Mol. Ther. Methods Clin. Dev. *15*, 257–263.
59. Subramanian, S., Maurer, A.C., Bator, C.M., Makhov, A.M., Conway, J.F., Turner, K.B., Marden, J.H., Vandenberghe, L.H., and Hafenstein, S.L. (2019). Filling adeno-associated virus capsids: Estimating success by cryo-electron microscopy. Hum. Gene Ther. *30*, 1449–1460.
60. Burnham, B., Nass, S., Kong, E., Mattingly, M., Woodcock, D., Song, A., Wadsworth, S., Cheng, S.H., Scaria, A., and O'Riordan, C.R. (2015). Analytical ultracentrifugation as an approach to characterize recombinant adeno-associated viral vectors. Hum. Gene Ther. Methods *26*, 228–242.
61. Fuerstenau, S.D., and Benner, W.H. (1995). Molecular weight determination of megadalton DNA electrospray ions using charge detection time-of-flight mass spectrometry. Rapid Commun. Mass Spectrom. *9*, 1528–1538.
62. Pierson, E.E., Keifer, D.Z., Selzer, L., Lee, L.S., Contino, N.C., Wang, J.C., Zlotnick, A., and Jarrold, M.F. (2014). Detection of late intermediates in virus capsid assembly by charge detection mass spectrometry. J. Am. Chem. Soc. *136*, 3536–3541.
63. Doussineau, T., Désert, A., Lambert, O., Taveau, J.-C., Lansalot, M., Dugourd, P., Bourgeat-Lami, E., Ravaine, S., Duguet, E., and Antoine, R. (2015). Charge detection mass spectrometry for the characterization of mass and surface area of composite nanoparticles. J. Phys. Chem. C *119*, 10844–10849.
64. Harper, C.C., Elliott, A.G., Oltrogge, L.M., Savage, D.F., and Williams, E.R. (2019). Multiplexed charge detection mass spectrometry for high-throughput single ion analysis of large molecules. Anal. Chem. *91*, 7458–7465.
65. Keifer, D.Z., Pierson, E.E., and Jarrold, M.F. (2017). Charge detection mass spectrometry: weighing heavier things. Analyst (Lond.) *142*, 1654–1671.
66. Kohlbrenner, E., Aslanidi, G., Nash, K., Shklyaev, S., Campbell-Thompson, M., Byrne, B.J., Snyder, R.O., Muzyczka, N., Warrington, K.H., Jr., and Zolotukhin, S. (2005). Successful production of pseudotyped rAAV vectors using a modified baculovirus expression system. Mol. Ther. *12*, 1217–1225.
67. Fernandez de la Mora, J. (2000). Electrospray ionization of large multiply charged species proceeds via Dole's charged residue mechanism. Anal. Chim. Acta *406*, 93–104.
68. Keifer, D.Z., Motwani, T., Teschke, C.M., and Jarrold, M.F. (2016). Acquiring structural information on virus particles with charge detection mass spectrometry. J. Am. Soc. Mass Spectrom. *27*, 1028–1036.
69. Horowitz, E.D., Rahman, K.S., Bower, B.D., Dismuke, D.J., Falvo, M.R., Griffith, J.D., Harvey, S.C., and Asokan, A. (2013). Biophysical and ultrastructural characterization of adeno-associated virus capsid uncoating and genome release. J. Virol. *87*, 2994–3002.
70. Bennett, A., Patel, S., Mietzsch, M., Jose, A., Lins-Austin, B., Yu, J.C., Bothner, B., McKenna, R., and Agbandje-McKenna, M. (2017). Agbandje-McKenna. M. Thermal stability as a determinant of AAV serotype identity. Mol. Ther. Methods Clin. Dev. *6*, 171–182.
71. Bernaud, J., Rossi, A., Fis, A., Gardette, L., Aillot, L., Büning, H., Castelnovo, M., Salvetti, A., and Faivre-Moskalenko, C. (2018). Characterization of AAV vector particle stability at the single-capsid level. J. Biol. Phys. *44*, 181–194.
72. Xu, Y., Guo, P., Zhang, J., Chrzanowski, M., Chew, H., Firrman, J.A., Sang, N., Diao, Y., and Xiao, W. (2020). Effects of thermally induced configuration changes on rAAV genome's enzymatic accessibility. Mol. Ther. Methods Clin. Dev. *18*, 328–334.
73. Aloor, A., Zhang, J., Gashash, E.A., Parameswaran, A., Chrzanowski, M., Ma, C., Diao, Y., Wang, P.G., and Xiao, W. (2018). Site-specific N-glycosylation on the AAV8 capsid protein. Viruses *10*, 644.
74. Galibert, L., Savy, A., Dickx, Y., Bonnin, D., Bertin, B., Mushimiyimana, I., van Oers, M.M., and Merten, O.-W. (2018). Origins of truncated supplementary capsid proteins in rAAV8 vectors produced with the baculovirus system. PLoS ONE *13*, e0207414.
75. Belyi, V.A., and Muthukumar, M. (2006). Electrostatic origin of the genome packing in viruses. Proc. Natl. Acad. Sci. USA *103*, 17174–17178.
76. Garmann, R.F., Comas-Garcia, M., Koay, M.S.T., Cornelissen, J.J.L.M., Knobler, C.M., and Gelbart, W.M. (2014). Role of electrostatics in the assembly pathway of a single-stranded RNA virus. J. Virol. *88*, 10472–10479.
77. Nam, H.-J., Gurda, B.L., McKenna, R., Potter, M., Byrne, B., Salganik, M., Muzyczka, N., and Agbandje-McKenna, M. (2011). Structural studies of adeno-associated virus serotype 8 capsid transitions associated with endosomal trafficking. J. Virol. *85*, 11791–11799.
78. Moullier, P., and Snyder, R.O. (2008). International efforts for recombinant adeno-associated viral vector reference standards. Mol. Ther. *16*, 1185–1188.
79. Ayuso, E., Blouin, V., Lock, M., McGorray, S., Leon, X., Alvira, M.R., Auricchio, A., Bucher, S., Chtarto, A., Clark, K.R., et al. (2014). Manufacturing and characterization of a recombinant adeno-associated virus type 8 reference standard material. Hum. Gene Ther. *25*, 977–987.
80. Contino, N.C., and Jarrold, M.F. (2013). Charge detection mass spectrometry for single ions with a limit of detection of 30 charges. Int. J. Mass Spectrom. *345–347*, 153–159.
81. Draper, B.E., Anthony, S.N., and Jarrold, M.F. (2018). The FUNPET—A new hybrid ion funnel-ion carpet atmospheric pressure interface for the simultaneous transmission of a broad mass range. J. Am. Soc. Mass Spectrom. *29*, 2160–2172.
82. Draper, B.E., and Jarrold, M.F. (2019). Real-time analysis and signal optimization for charge detection mass spectrometry. J. Am. Soc. Mass Spectrom. *30*, 898–904.

OMTM, Volume *23*

Supplemental information

Quantitative analysis of genome packaging in recombinant AAV vectors by charge detection mass spectrometry

Lauren F. Barnes, Benjamin E. Draper, Yu-Ting Chen, Thomas W. Powers, and Martin F. Jarrold

Analysis of Alkaline Gels and Comparison to CDMS results

BioRad Image Lab 5.2.1 (version 6.0.1.34) software was used to perform a densitometry analysis of the alkaline gels. The gel and CDMS signals can be divided into three main subclasses: the high mass peak or band at close to the packaging capacity, the GOI peak or band, and the low intensity, low mass tail that extends to the empty capsid in some of the CDMS spectra. The analog of the CDMS low mass tail was absent or small in the gels and so we focused on the high mass band and the GOI band for the gels. The relative integrated intensities are summarized in Table S1. We caution that the features often overlap and the locations of the dividing lines between them is somewhat subjective.

The gels and CDMS measurements have different sensitivities. The signals from bands on a gel are proportional to the number of DNA bases present while for CDMS the signal is proportional to the number of molecules. Thus the gels are more sensitive to longer DNA molecules and this explains why the low mass tail evident in the CDMS measurements is absent or small in the gels. To enable a quantitative comparison between the gels and the CDMS measurements we corrected for the sensitivity difference by multiplying the CDMS signals by the DNA mass given by the measured mass minus the mass of the empty particle. We then integrated the signals in the GOI peaks and the high mass peaks. The fraction of the signal in the GOI peak (the integrated signal for the GOI peak divided by the sum of the integrated signals the high mass and GOI peaks) is shown in Table S2 which also includes the corresponding fraction of the GOI band from the gels.

Figure S1 shows a plot of the GOI fraction obtained from the gels versus the GOI fraction obtained from the adjusted CDMS measurements. The dashed line in the figure shows the result where the GOI fraction from the gels and CDMS measurements are identical. The relative intensities from the gels are in most cases slightly higher than the relative intensities from the CDMS measurements. The two measurements are completely different so the good agreement between them is gratifying.

Table S1. Table showing the percentages of the signal in the high mass peak or band, GOI peak or band, and the low mass tail for the GOIs studied here. For the gels the low mass tail was small or missing and it is not included. Results are shown for alkaline gels with (+) and without (−) DNase I treatment.

	Alkaline Gel (−)		Alkaline Gel (+)		CDMS		
GOI	High Mass Band	GOI Band	High Mass Band	GOI Band	High Mass Peak	GOI Peak	Low Mass Tail
CMV-CRE	33	67	38	62	26	64	10
CMV-GFP	39	61	48	52	32	49	19
CMV-mcherry	37	63	34	66	22	68	9
CAG-GFP	35	65	39	61	20	58	22
CAG-mcherry	32	68	38	62	25	62	13
EF1a-GFP	25	75	26	74	18	58	25
CBA-GFP	-	100	-	100	-	72	28
CMV-SaCas9	-	100	-	100	-	52	48

Table S2. Table showing the fractions in the GOI peaks or bands for the different GOIs. The fractions were determined from the integrated intensity of the GOI peak or band divided by the sum of the integrated intensities of the high mass and GOI peaks or bands. Results are shown for the alkaline gel, with (+) and without (−) DNase I treatment. The results for the CDMS measurements have been adjusted as described above to account for the sensitivity of the gels to DNA length.

GOI	Fraction in GOI Peak or Band		
	Gel (−)	Gel (+)	CDMS
CMV-CRE	0.67	0.62	0.57
CMV-GFP	0.61	0.52	0.48
CMV-mcherry	0.63	0.66	0.63
CAG-GFP	0.65	0.61	0.63
CAG-mcherry	0.68	0.62	0.62
EF1a-GFP	0.75	0.74	0.72

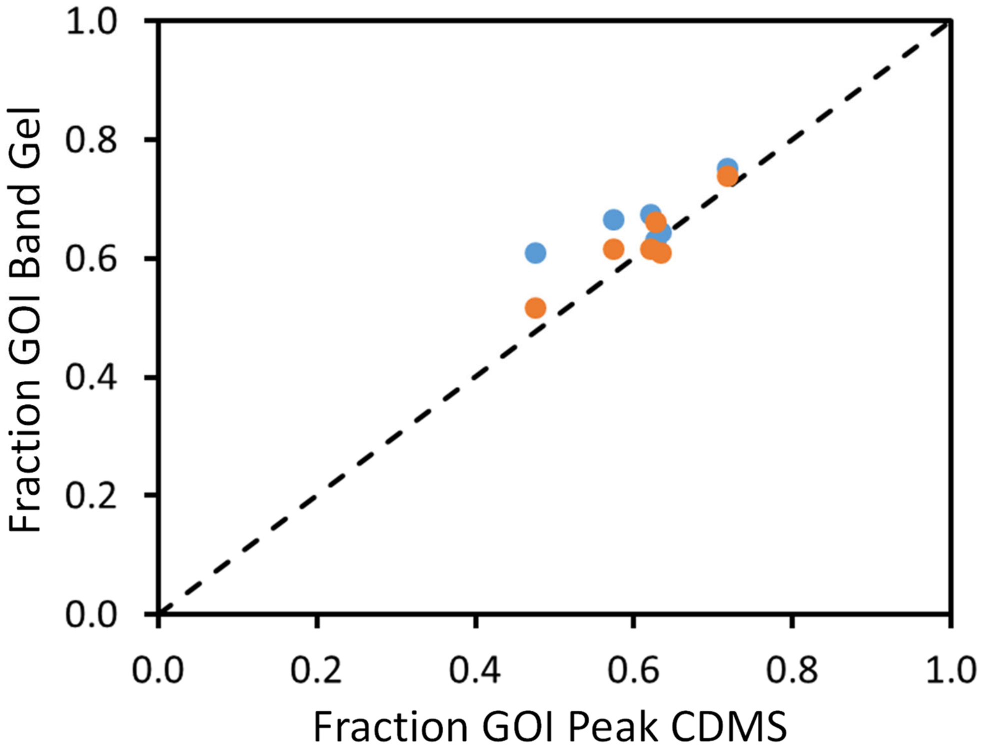

Figure S1. Plot of the relative intensity of the GOI peak or band determined from the gels against the same quantity determined by CDMS. The orange points are for gels with DNase I the blue points are without. The dashed line shows the result where the gels and CDMS give the same fractions.

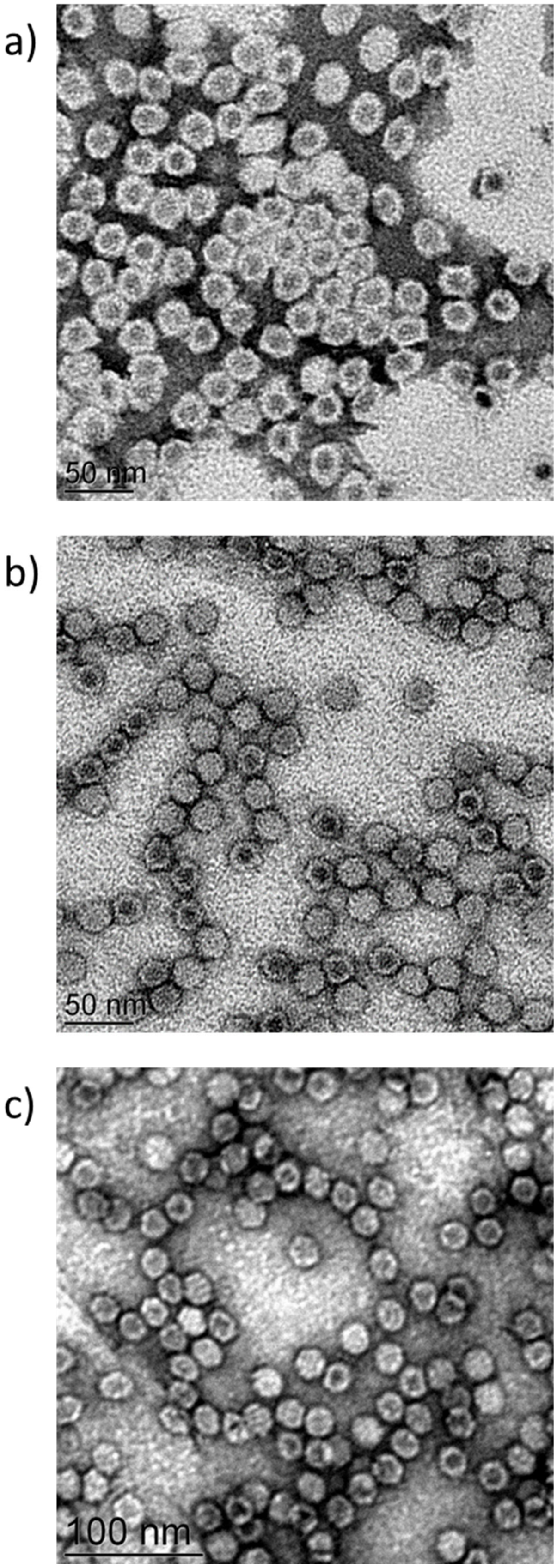

Figure S2. Representative TEM images for Virovek vectors. a) empty AAV8; b) CMV-SaCas9 (4.84 kb); and c) CMV-CRE (2.22 kb). In a) stain has penetrated most of the particles (they have a darkened center) indicating that they are empty. CDMS measurements for this sample indicate that all the particles are empty. A few particles in the image, particularly near the top, are not stained, presumably because the stain did not penetrate. In b) most of the particles have not picked up stain suggesting that they are full. The CDMS measurements for CMV-SaCas9 show a peak for the GOI near the packaging capacity and a broad low mass tail (comprising around 48% of the signal) that extends almost to the mass of the empty capsid. The stained particles in this case must have partial genomes. In c) there is a heterogeneous mixture where some particles are not stained (full), some are lightly stained (partial), and some are heavily stained (empty). The CDMS measurements for CMV-CRE show a peak at close to the packaging capacity, a peak for the GOI at intermediate masses and low mass tail that extends to the empty capsid mass. Overall, the CDMS measurements provide a more robust characterization of the genome content than negative stain EM.
